# Editorial: Molecular mechanisms and precision medicine in rare diseases

**DOI:** 10.3389/fmolb.2026.1778169

**Published:** 2026-01-19

**Authors:** Hector Rodriguez Cetina Biefer, Abdallah Elkhal, Mohamed Taha Moutaoufik

**Affiliations:** 1 Department of Cardiac Surgery, University Hospital Zürich, Zurich, Switzerland; 2 NAD+ Immunology Laboratory, Cardiovascular Department, Huntington Medical Research Institutes, Pasadena, CA, United States; 3 Faculty of Medical Sciences, UM6P Hospitals, Mohammed VI Polytechnic University, Ben Guerir, Morocco

**Keywords:** biochemical pathway, biochemistry, cellular models, diagnosis, omics, personalized medicine, rare diseases, therapeutic targets

Rare diseases represent a substential public health burden affecting millions of individuals worldwide. Rare disorders often present with phenotypic variability, limited clinical awareness and co-phenotypes with more prevalent diseases. In contrast, many rare diseases arise from well-defined pathogenic lesions, rendering them prototypical candidates for precision and personalized biomedical approaches. Technological and conceptual advances in molecular biology, together with refined diseases-specific models, have fundamentally reshaped our ability to investigate diseases that were previously regarded as be poorly understood.

The Research Topic “Molecular Mechanisms and Precision Medicine in Rare Diseases” comprises six contributions that are relevant and complementary in showing the utility of molecular research for the diagnosis and understanding of diseases, as well as the application of this knowledge for the treatment of diseases that are considered rare.

A common problem in rare diseases is the presence of diagnostic uncertainty due to the heterogeneity observed in the clinical case. The report by Yang et al. on erythropoietic protoporphyria (EPP) is one such example, wherein the patient has an uncommon form of heme biosynthesis deficiency. In this study, Yang et al. use a very detailed pathological analysis along with genome analysis to identify a splicing mutation within the FECH gene, thus resolving the uncommon case presentation in this patient.

In addition to diagnosis, a detailed understanding the underlying mechanisms of the cellular and tissue-level mechanisms is critical for leveraging genetic information as a foundation for therapeutic rationale. This issue is addressed in a study by Maqoud et al. investigating Cantύ syndrome caused by gain-of-function mutation in ATP-sensitive potassium (KATP) channels. Using a mouse model, the authors demonstrate that aberrant KATP channel activity disrupts tight junctions integrity in the colonic epithelium. These findings refine the mechanistic understanding of Cantύ syndrome and offer immportant insight into therapeutic strategies.

Precision medicine in rare diseases requires the integration of molecular diagnostics with rigorous evaluation of long-term clinical outcomes. Choi and Yang contribute to this field through a cohort study examining the long-term outcome of growth hormone therapy in patients with Prader-Willi syndrome. By assessing mortality and metabolic outcome, including the risk of developing type 2 diabetes, their work addresses a critical gap in real-world evidence informing individualized therapeutic strategies.

The diagnostic complexity introduced by phenocopies is further illustrated by the study of Zhao et al. on pseudohypoparathyroidism type 1B, a disorder that shares overlapping clinical and biochemical features with Gitelman syndrome. Through comprehensive biochemical and molecular analysis, the authors delineate key distinctions that differentiate hormone resistance syndromes from primary renal tubular disorders underscoring the importance of accurate diagnosis of rare endocrine disorders.

Robust Disease-relevant models must be well characterized and sufficiently powerful to enable mechanistic interrogation and therapeutic development. Schiffmaier et al. address this need by generating human periodontal ligament cells harboring ALPL mutations, thereby establishing patient-relevant models of hypophosphatasia models. These models faithfully tissue-specific mineralization defects and provide a valuable platform for mechanistic studies and drug screening. This approach underscores the importance of precise and disease-pecific cellular models in accelerating the process of translational research for rare skeletal and dentofacial disorders.

Notably, this Research Topic broadens the scope of precision medicine beyond host genetics alone. Suryavanshi et al. investigate rare microbial phylotypes across stone, urine, and stool specimens in urinary stone disease, identifying associations between microbial biomarkers and disease susceptibility. Although urinary stone disease is not classically defined as a rare genetic disorder, these findings highlight the potentially contribution of rare microbial communities to disease susceptibility and progression.

The six articles included in this Research Topic converge on several key overarching messages. First, integrated molecular diagnostics grounded in genetics, biochemistry, and pathology are critical cornerstone for the accurate diagnosing of rare diseases. Second, mechanistic basic research based using cellular and tissue-based models is central in harnessing genetic variability and translate it into rational therapeutic strategy. Third, precision therapies often rely on longitudinal follow-up to rigorously evaluate treatment efficacy and safety within well-defined genetic populations. Finally, precision medicine approaches increasingly benefit from the integration of multi-omics strategies, such as microbiome profiling, to gain deeper insights into disease pathology.

In conclusion, this Research Topic demonstrates that methodological rigor, mechanistic insights, and translational relevance can be seamlessly integrated to advance precision medicine in rare diseases. By linking diagnostic innovation with disease mechanisms and clinical outcomes, this Research Topic of articles outlines a clear path forward towards improved accuracy and the development on individualized therapeutic strategies to better serve patients with rare diseases.

